# Effect of Strong Opinions on the Dynamics of the Majority-Vote Model

**DOI:** 10.1038/s41598-018-26919-y

**Published:** 2018-06-07

**Authors:** André L. M. Vilela, H. Eugene Stanley

**Affiliations:** 1 Universidade de Pernambuco, Recife, PE 50100-010 Brazil; 2 Boston University, Center for Polymer Studies and Department of Physics, Boston, MA 02215 USA

## Abstract

We study how the presence of individuals with strong opinions affects a square lattice majority-vote model with noise. In a square lattice network we perform Monte-Carlo simulations and replace regular actors *σ* with strong actors *μ* in a random distribution. We find that the value of the critical noise parameter *q*_*c*_ is a decreasing function of the concentration *r* of strong actors in the social interaction network. We calculate the critical exponents *β*/*ν*, *γ*/*ν*, and 1/*ν* and find that the presence of strong actors does not change the Ising universality class of the isotropic majority-vote model.

## Introduction

In recent years, spin systems with short range interactions have been used to study the collective behavior found in opinion formation. The majority-vote model with noise has been a popular approach to investigating social interaction dynamics in regular and complex networks^1–17^. In its isotropic version, each node of an square lattice network is assigned a spin variable with either of two values, ±1, which represent an individual’s opinion in the network. At some time *t* an individual adopts the majority sign of the spins in its neighborhood with a probability 1 − *q* and the minority sign with a probability *q*. This probability *q* is the noise parameter of the model and quantifies the social temperature. Increasing *q* promotes the formation of opposite-opinion pairs in the social interaction network. This system undergoes a second-order phase transition when the critical noise reaches *q*_*c*_ ≈ 0.075 and enters the same universality class as the equilibrium two-dimensional Ising model with critical exponents *β*/*ν* ≈ 0.125, *γ*/*ν* ≈ 1.75, and 1/*ν* ≈ 1.0^2,3^.

The powerful computers now available allow us to analyze the behavior of systems composed of millions of interacting elements. Focusing this computational power on the critical behavior of the majority-vote model has yielded numerous extensions. Among these are agent differentiation and diffusion^4,5^, three state systems^6–8^, and complex noise distributions^9–12^. The development of complex network theory and its expansion across numerous scientific fields has also contributed to the investigation of this social model. Recently majority-vote dynamics were studied in several complex networks, including small-world networks, random graphs, and scale-free networks^13–19^. Many expanded and modified versions of the majority-vote model in these complex networks belong to new universality classes, and some present critical exponents that are Ising-like, supporting the conjecture by Grinstein *et al*.^20^. This conjecture states that irreversible systems with up-down symmetry belong in the universality class of the equilibrium Ising model for regular square lattices.

Here we investigate how individuals with strong opinions affect the critical behavior of the majority-vote model. Those agents possess a greater spin variable and represent individuals which its opinion prevails in a social network, such as scientists, politicians, religious leaders or family members. We use Monte Carlo simulations and finite-size scaling techniques to calculate the critical noise parameter *q*_*c*_ and to obtain the phase diagram of the system. We also perform computer simulations to estimate the critical exponents *β*/*ν*, *γ*/*ν*, and 1/*ν* as a function of the concentration in the network of individuals with strong opinions.

We organize this presentation as follows. In Sec. The Model we describe the majority-vote model with strong opinions and introduce the relevant quantities used in our computational analysis. In Sec. Results and Discussion we describe our numerical results, and in Sec. Conclusions and Final Remarks we present our conclusions.

## The Model

The majority-vote model with strong opinions utilizes a set of individuals in a geometric network of social interactions. The opinions of these individuals are represented by two numerical variables: *σ* = ±1.0 and *μ* = ±1.5, which are randomly distributed in the network. Here we place the individuals in the nodes of a regular two-dimensional square lattice of size *N* = *N*_*σ*_ + *N*_*μ*_, where *N*_*σ*_ and *N*_*μ*_ are the numbers of agents of *σ* and *μ* kind, respectively. We define *r* = *N*_*μ*_/*N* to be the relative concentration of individuals with strong opinions *μ* and one can recover the isotropic majority-vote model by using *r* = 0 or *r* = 1^2^. For the symmetry of this model, we use 0 ≤ *r* ≤ 1/2.

The dynamics of the system resemble those of the standard majority-vote model. We randomly select a spin *α*_*i*_ and determine the majority opinion among the neighboring spins. Here *α*_*i*_ is a generic spin that is equal to either *σ*_*i*_ or *μ*_*i*_. Then the variable of spin *α*_*i*_ is flipped with a probability

1 $$w({\alpha }_{i})=\frac{1}{2}[1-(1-2q)\,{\rm{s}}{\rm{g}}{\rm{n}}\,({\alpha }_{i})\,{\rm{s}}{\rm{g}}{\rm{n}}\,(\sum _{\delta =1}^{{k}_{i}}\,{\alpha }_{i+\delta })],$$

where sgn(*x*) = +1, 0, −1 in case *x* < 0, *x* = 0 and *x* > 0, respectively. The sum runs over all *k*_*i*_ nodes attached to the spin *α*_*i*_ and for regular square lattice networks *k*_*i*_ = 4 for all *i* ∈ *N*. With probability *q* the selected spin adopts the sign opposite to that of the majority of its neighbors, and with probability 1 − *q* adopts the same sign. During each Monte Carlo step this procedure is repeated *N* times, so that on average each network site can flip its signal once per Monte Carlo step. The control variable *q* is the noise parameter of spin *α*_*i*_, and increasing *q* decreases the probability that *α*_*i*_ will agree with its local majority.

**Figure 1:**
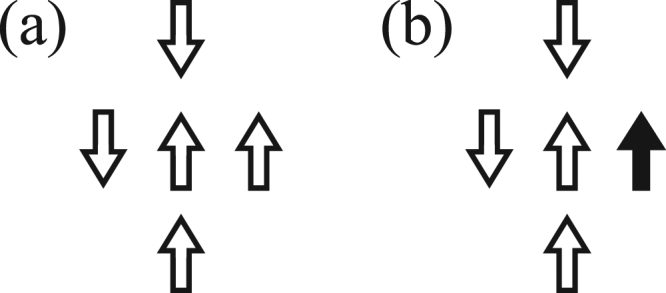
Illustration of a “tie” for a randomly selected site *σ*_*i*_. Open (closed) arrows represents *σ* (*μ*) individuals in the network. In (**a**) there is a lack of majority and the probability of flip is independent of *q* and equal 0.5. In (**b**) we have a tie for the signs of the spins, two positive and two negative opinions, but we do have a majority of it because the presence of a strong opinion *μ*.

In this work, a strong opinioned *μ* agent has a stronger influence in the network of social interactions and for simplicity, we assume that its spin changes under the same probabilities of a regular individual *σ*. In other words, both types of agents are equally susceptible to flip their opinion under the majority-vote rules. As a consequence of this assumption, we observe that the consensus of the system is weakened by increasing the concentration *r*, since flipping a strong agent produces a stronger effect on the social network than flipping a regular agent. In particular, for a system where all sites are initially pointing up and *r* = 0, increasing *q* above zero produces new configurations and ties may occur. When there is no majority opinion among the neighbors in this case, the flip probability of the central site is unaffected by noise parameter *q*. To illustrate this, Fig. 1(a) shows that the flip probability is *w*(*σ*_*i*_) = 0.5, and we randomly select an opinion for central site *σ*_*i*_. Figure 1(b) shows a similar configuration with one *μ* individual present (*r* ≠ 0) and, in this case, we introduce new possible ties in the system. The signs of the spins of the neighborhood of *σ*_*i*_ are tied, but there is a positive majority. Thus, we find that *w*(*σ*_*i*_) = *q* and that the opinion of the spin *σ*_*i*_ is influenced by *q*, the social temperature of the network, which lowers the consensus of the system. We observe that the model experiences a phase transition when the critical value of the social temperature *q*_*c*_ is reached.

To investigate how the presence of strong opinions affects the dynamics of the system, we first calculate the average opinion defined by

2 $$m=\frac{1}{{N}_{\sigma }+3{N}_{\mu }/2}|\sum _{i=1}^{N}\,{\alpha }_{i}|,$$

we next examine three quantities that depend on the noise parameter *q*, on the relative concentration of strong opinions *r*, and on system size $L=\sqrt{N}$,

3 $$M(q,r,L)={\langle {\langle m\rangle }_{t}\rangle }_{c},$$

4 $$\chi (q,r,L)={L}^{2}[{\langle {\langle {m}^{2}\rangle }_{t}\rangle }_{c}-{\langle {\langle m\rangle }_{t}\rangle }_{c}^{2}],$$

and

5 $$U(q,r,L)=1-\,\frac{{\langle {\langle {m}^{4}\rangle }_{t}\rangle }_{c}}{3{\langle {\langle {m}^{2}\rangle }_{t}\rangle }_{c}^{2}},$$

where *M*, *χ*, and *U* are magnetization, magnetic susceptibility, and the Binder fourth-order cumulant, respectively. The time averages in the stationary regime are 〈…〉_*t*_, and the configurational averages are 〈…〉_*c*_. The characteristics of the critical behavior of the model are investigated using computer simulations and finite-size scaling analysis.

## Results and Discussion

We perform Monte Carlo simulations on lattices with linear sizes ranging from *L* = 5 to 140 and with periodic boundary conditions. For each value of noise *q*, we set all spins to point up, i.e., *σ*_*i*_ = +1.0 and *μ*_*j*_ = +1.5 for all *i* and *j* in the network. We next perform 5 × 10^4^ time steps in the simulation to overcome transients and allow the system to reach a steady state. The time averages were estimated from the next 5 × 10^5^ Monte Carlo steps. To calulate the configurational averages we repeat the simulations up to 120 independent samples.

**Figure 2:**
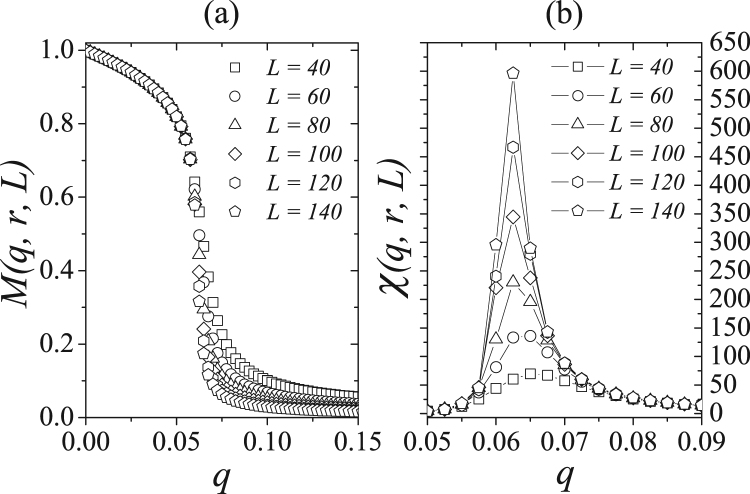
(**a**) Magnetization and (**b**) susceptibility versus the noise parameter *q* for the relative presence of strong opinions *r* = 0.1 and several values of the system size *L*. In (a) the finite-size effects are observed for *q* near and above the critical value. In this region, the magnetic susceptibility exhibit a peak which amplitude and position depend on the system size.

**Figure 3:**
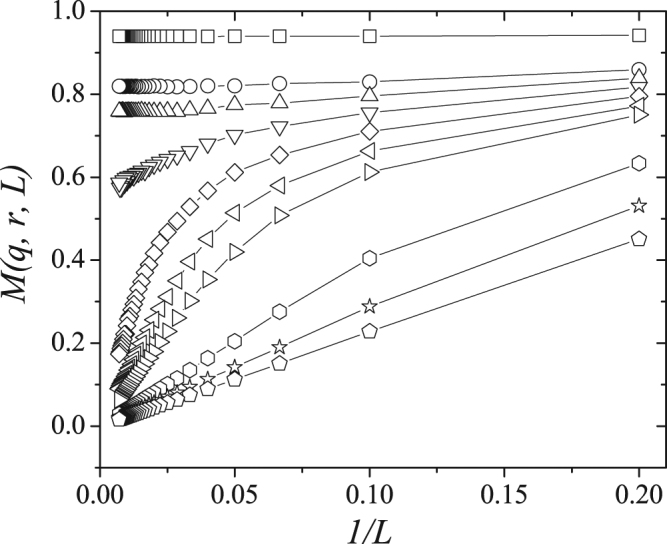
Magnetization for *r* = 0.1 as a funcion of 1/*L* for several values of the noise parameter *q* and *L* ranging from 5 up to 140. From top to bottom we have *q* = 0.025, 0.050, 0.055, 0.060, 0.065, 0.070, 0.075, 0.100, 0.125 and 0.150. Lines are just a guide to the eyes.

For the level of strong opinions *r* = 0.1 present in the network, Fig. 2 shows the dependence of magnetization *M*(*q*, *r*, *L*) and susceptibility *χ*(*q*, *r*, *L*) on system size *L*. Note that the system undergoes a phase transition from ordered to disordered at some critical value *q*_*c*_, where the magnetic susceptibility *χ*(*q*, *r*, *L*) exhibits a maximum. Note that *q*_*c*_ is dependent on system size *L*. Figure 2(a) shows that the magnetization goes to zero when *q* > *q*_*c*_ in the thermodynamic limit *L* → ∞. Figure 3 shows the magnetization versus the inverse of the system size for several values of noise parameter *q* for *r* = 0.1. Note that the system undergoes a phase transition for *q* near 0.065, where the magnetization goes to zero when *L* increases.

**Figure 4:**
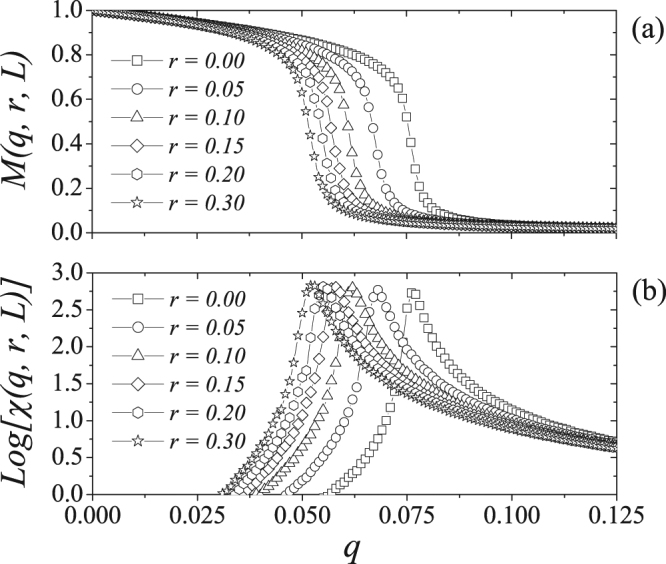
(**a**) Magnetization and (**b**) susceptibility versus the noise parameter *q* for several values of the relative presence of individuals with strong opinions *r* for *L* = 140. We can see that increasing of the relative presence *r* the critical value for *q* decreases. Lines are a guide to the eyes.

Figure 4 shows the magnetization *M*(*q*, *r*, *L*) and the logarithm of the magnetic susceptibility *χ*(*q*, *r*, *L*) with *L* = 140 for several values of the presence *r* of strong-opinioned individuals. Note that the system undergoes a phase transition from ordered to disordered at some critical value *q*_*c*_, which is dependent on *r*. We also note that the critical noise parameter is a decreasing function of the relative concentration *r*. This indicates that the presence of this type of agents in the network weakens the consensus of the system. The pseudocritical noise values *q*_*c*_(*L*) are located near the peaks of the susceptibility *χ*(*q*, *r*, *L*) and support the results obtained for *M*(*q*, *r*, *L*).

**Figure 5:**
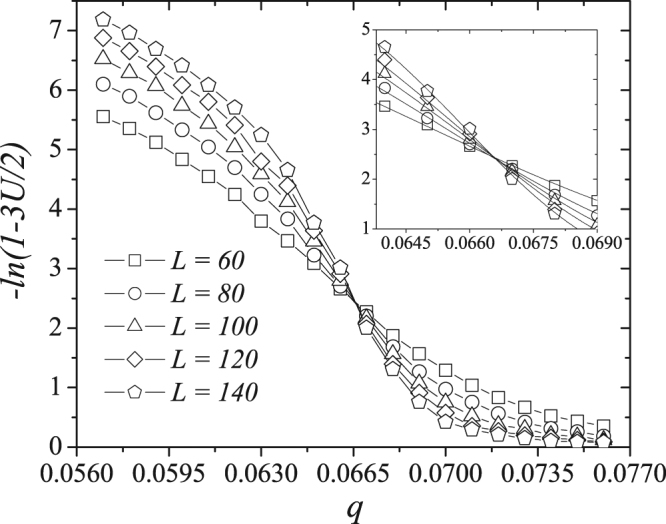
The Binder fourth-order cumulant *U*(*q*, *r*, *L*) as a function of the noise parameter *q* for the majority-vote model with strong opinions with *r* = 0.05 and several system sizes. The critical noise for this value of relative presence was found to be *q*_*c*_ = 0.0665 ± 0.0003 and was obtained in the curves intersection point. In the inset we exhibit the details of the interception for different system sizes and a cubic fit for the data points in this region.

Figure 5 shows the dependence of the Binder fourth-order cumulant *U*(*q*, *r*, *L*) on the noise parameter *q* for *r* = 0.05 and for different values of *L*. Note that the curves of the different system sizes intercept when 0.063 < *q* < 0.070. Figure 5 (inset) shows a magnification of the Binder cumulant data and its polynomial fit in the region near the curve interception, where *q*_*c*_ does not depend on the system size. Here we estimate the critical noise to be *q*_*c*_ = 0.0665 ± 0.0003 when *r* = 0.05.

**Figure 6:**
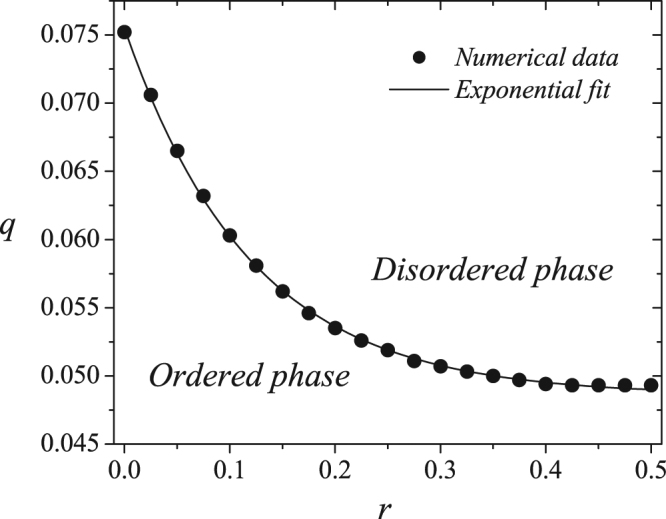
Phase diagram of the majority-vote model with strong opinions in the plane *q* versus *r*, separating the ordered and the disordered phases of the system. The points are the numerical values of the critical noise parameter *q*_*c*_ estimated from the simulations, where error bars are shorter than the size of the symbols. The line is an exponential fit for the critical noise parameter.

We calculate the Binder fourth-order cumulant *U*(*q*, *r*, *L*) for other *r* values and Fig. 6 shows the phase diagram of the model. Here the symbols represent the critical values of the noise parameter *q*_*c*_ estimated using Monte Carlo simulations, and the line is an exponential fit for the numerical results. Here we obtain ${q}_{c}(r)={c}_{1}+{c}_{2}{e}^{-r/{r}_{0}}$, where *c*_1_ = 0.0486(1), *c*_2_ = 0.0269(1), and *r*_0_ = 0.119(1). Table 1 provides the critical noise values for each concentration *r* in the model. Note that the critical value *q*_*c*_ is a decreasing function of the relative presence of strong opinions *r*.

**Table 1:** The critical noise *q*_*c*_ and its error versus the concentration of strong opinions *r*.

*r*	0.00	0.05	0.10	0.15	0.20	0.25	0.30	0.35	0.40	0.45	0.50
*q*	0.0752	0.0665	0.0603	0.0562	0.0535	0.0519	0.0507	0.0500	0.0494	0.0493	0.0493
*q*_*err*_	0.0002	0.0003	0.0004	0.0001	0.0003	0.0001	0.0001	0.0001	0.0001	0.0001	0.0001

We can obtain three critical model exponents because near the critical noise *q*_*c*_, the pseudocritical noise, the magnetization, the susceptibility, and the Binder cumulant satisfy the finite-size scaling relations^21^

6 $${q}_{c}(L)={q}_{c}+b{L}^{-1/\nu },$$

7 $${U}_{L}(q,r)=\tilde{U}(\varepsilon {L}^{1/\nu }),$$

8 $${M}_{L}(q,r)={L}^{-\beta /\nu }\tilde{M}(\varepsilon {L}^{1/\nu }),$$

and

9 $${\chi }_{L}(q,r)={L}^{\gamma /\nu }\mathop{\chi }\limits^{ \sim }(\varepsilon {L}^{1/\nu }),$$

where *ε* = *q* − *q*_*c*_ is the distance to the critical noise, *b* is a constant, and $\tilde{M}$, $\tilde{\chi }$, and $\tilde{U}$ are scaling functions that only depend on the scaled variable *x* = *εL*^1/*ν*^.

**Figure 7:**
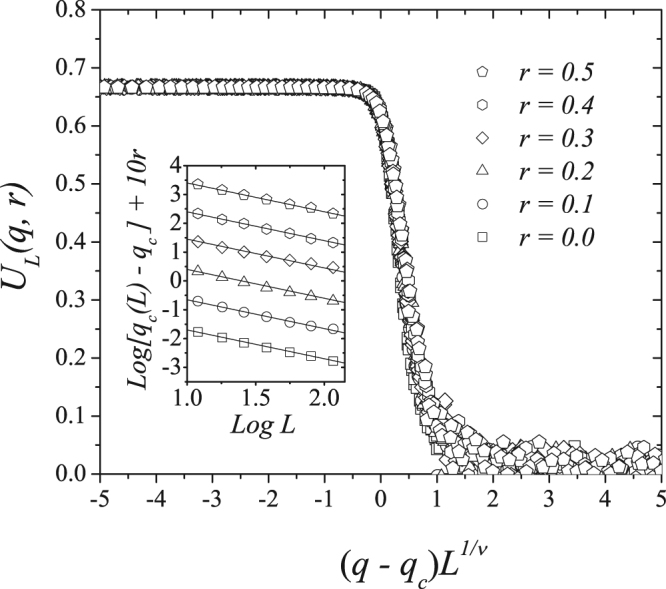
Scaling plot of the Binder fourth-order cumulant *U*_*L*_(*q*, *r*) for five system sizes *L* = 60, 80, 100, 120 and 140, with *r* = 0.0, 0.1, 0.2, 0.3, 0.4 and 0.5. In this result we used 1/*ν* = 1 and all data collapsed in only one universal curve. In the inset we exhibit the the plot of Log[*q*_*c*_(*L*) − *q*_*c*_] versus Log(*L*^−1^), giving us the estimate of 1/*ν* ≈ 1.0 for all *r*.

Using Eq. (6), we estimate the critical exponent 1/*ν* by plotting the logarithm of the distance between the pseudocritical and the critical noise *q*_*c*_(*L*) − *q*_*c*_ versus the logarithm of *L*^−1/*ν*^. We obtain 1/*ν* ≈ 1.0 for all values of *r*. In Fig. 7 we show the scaling plot of the the Binder cumulant for 1/*ν* = 1.0, with different system sizes and concentrations, yielding a universal curve for *U*_*L*_(*q*, *r*).

**Figure 8:**
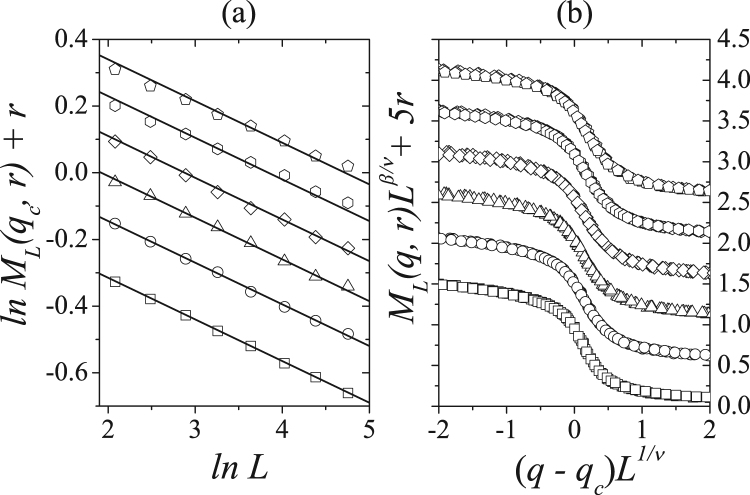
(**a**) Magnetization as a function of linear system sizes *L* in log-log scale for several concentrations *r* with *q* = *q*_*c*_(*r*). (**b**) Rescaling for the magnetization versus the rescaled noise parameter for *L* = 60, 80, 100, 120 and 140 and several values of the concentration *r*. Here, we used *β*/*ν* = 0.125 and 1/*ν* = 1.0. From bottom to the top we have *r* = 0.0, 0.1, 0.2, 0.3, 0.4 and 0.5.

Figure 8(a) shows the logarithm of the magnetization calculated using Eq. (3) versus the logarithm of system size *L* for *q* = *q*_*c*_(*r*). From bottom to top *r* = 0.0, 0.1, 0.2, 0.3, 0.4, and 0.5. We use the linear coefficient of each line to estimate the critical model exponents in which for each value of *r* and we find *β*/*ν* = 0.125, taking the error bars into consideration. The scaling functions for magnetization *M*_*L*_(*q*, *f*, *p*)*L*^*β*/*ν*^ versus rescaled noise parameter (*q* − *q*_*c*_)*L*^1/*ν*^ confirms this result, as shown in the data collapsed plotted in Fig. 8(b). Here we use 1/*ν* = 1.0 and each scaling plot is shifted up adequately to avoid overlapping between them.

**Figure 9:**
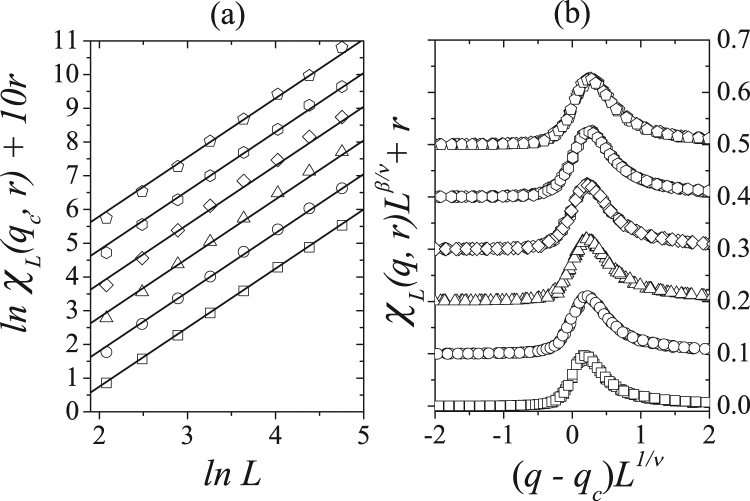
(**a**) Magnetic susceptibility as a function of linear system sizes *L* in log-log scale for several values of *r* with *q* = *q*_*c*_(*r*). (**b**) Rescaling for the susceptibility versus the rescaled noise parameter for *L* = 60, 80, 100, 120 and 140 and several values of the concentration *r*. From bottom to the top we have *r* = 0.0, 0.1, 0.2, 0.3, 0.4 and 0.5. In the latter plot, we used *γ*/*ν* = 1.75 and 1/*ν* = 1.0.

We perform a similar analysis and obtain the susceptibility as a function of system size for several values of *r* with *q* = *q*_*c*_(*r*). Figure 9(a) shows these results when we estimate the critical exponent *γ*/*ν* to be 1.75 for all *r* values. Using this result, we plot the dependence of the rescaled magnetic susceptibility *χ*_*L*_(*q*, *r*)*L*^−*γ*/*ν*^ on the rescaled noise (*q* − *q*_*c*_)*L*^1/*ν*^ using the critical noise parameters in Table 1, with *γ*/*ν* = 1.75 and 1/*ν* = 1.0. Other concentration values *r* exhibit the same features and the same qualitative results for the magnetization and susceptibility, indicating that a strong opinion majority-vote belongs to the Ising universality class.

## Conclusion and Final Remarks

We have investigated a majority-vote model with strong opinions using a regular two-dimensional square lattice of size *N* = *L* × *L*. Using this model, we consider a relative concentration *r* of individuals that have strong opinions in a social interaction network. These individuals hold an opinion that is 50% stronger than a regular Ising opinion variable. We find that the presence of this type of individual weakens the consensus of the network, which presents a second order phase transition from ordered to a disordered state. Performing Monte Carlo simulations, we analyze the critical behavior of the model on lattices with linear sizes up to *L* = 140. We find that the critical exponents of the model, *β*/*ν* ≈ 0.125, *γ*/*ν* ≈ 1.75 and 1/*ν* ≈ 1.0, are the same as those in an equilibrium two-dimensional Ising model and do not change with the concentration of strong opinions *r*. We note that the scaling plot of the magnetization and susceptibility showed in Figs 8 and 9 were shifted up to avoid overlapping, but they yield only one universal curve each, not depending on *r*, despite the different behaviors observed in Fig. 4. We conclude that the majority-vote model with strong opinions in a 2*D* square lattice belongs to the Ising universality class and this result agree with Grinstein criterion for nonequilibrium stochastic spin systems with up-down symmetries on regular lattices^20^.

Many variants of this model and its effects on the critical behavior might be considered, such as interactions mapped in complex or multilayer networks and other values for the spin variable of strong individuals.
